# 12 Tips for Pivoting to Teaching in a Virtual Environment

**DOI:** 10.15694/mep.2020.000170.1

**Published:** 2020-08-18

**Authors:** Benjamin Collins, Ryan Day, Joanne Hamilton, Kathleen Legris, Helen Mawdsley, Tanya Walsh

**Affiliations:** 1 University of Manitoba

**Keywords:** Health Professions Education, virtual learning, online assessments, faculty development, cognitive load theory, COVID-19

## Abstract

This article was migrated. The article was marked as recommended.

COVID-19 has necessitated a rapid shift to teaching in virtual environments across the educational spectrum. In this respect, instructors previously unfamiliar, or under-familiar, with virtual teaching environments need to learn quickly and effectively how these environments work and how they can be used to successfully deliver courses, especially within health professions education contexts. These twelve tips provide insight on the practice of teaching in virtual environments, from course design, to student engagement, to assessment practices, to maximising the potential that technology can provide for both the instructor and the students. Moreover, these tips inform virtual pedagogical practices in the health professions for all levels of experience.

## Introduction

The reality of the COVID-19 pandemic has necessitated a major and rapid shift in teaching strategies across health professions education and throughout educational programs globally. Specifically, the need for physical distancing has severely constrained traditional face-to-face lectures and teaching strategies, and encouraged a shift to online and virtual teaching strategies ( Carlson, 2020; DeFilippis, Stefanescu Schmidt and Reza, 2020; Prem *et al.*, 2020).

Virtual teaching environments present a unique set of opportunities and challenges that have been increasingly utilised over the past decade in health professions education for distance education and courses using blended learning models ( Bonk and Graham, 2012). The Office of Education and Faculty Development (OEFD) at the University of Manitoba provides resources and training for health sciences faculty to develop competency and expertise for delivering courses in virtual teaching environments. This knowledge base has been called upon for the rapid transition to teaching in virtual environments during the COVID-19 pandemic. Within this context, and drawing on OEFD’s experiences of faculty development and support during the COVID-19 affected rapid teaching transition in March and April 2020, we present 12 tips for teaching in virtual environments. We designed these tips for faculty who are both relatively new to virtual teaching, as well as those who have prior experience, with the latter potentially benefiting from new and different perspectives on the application and effects of teaching through technology presented below.

## Tip 1 – Review learning objectives to align with virtual environments

The development of online courses and programs should include careful consideration of the learning objectives. Wiggins and McTighe (2005) encourage educators to begin the design process by identifying what evidence is required for learners to demonstrate the desired level of proficiency before planning teaching and learning experiences, i.e. through constructive alignment ( Biggs, 2002). Within health sciences curricula, learning objectives are likely to be rigid, as programs must demonstrate professional competencies and standards set by accrediting bodies. Learning objectives will therefore remain in place, but the context in which they are performed will transform within virtual environments. Educators must think critically about what can realistically be achieved in a virtual environment, as, given the nature of the subject matter, clinical spaces are an imperative context that will be difficult to completely substitute. Pausing and reflecting on learning objectives is an important step in deciding what is feasible and how best to align learning objectives with the reality of teaching in virtual environments.

## Tip 2 – Review resources for teaching in a virtual environment

The interdependency between content, pedagogy, and technology is a unique characteristic of online teaching. Learning in a digital context happens through discussion, reflection, and collaboration with students who are prepared to engage in active learning with a community of peers. A wide range of tools and technologies is available, and choosing which to use is dependent on two key factors: its support of the teaching objectives, and its unique affordances and potential learning benefits ( Ng, 2015). For example, learning management systems are designed to foster social and collaborative learning in both synchronous and asynchronous settings; social media can enhance social presence and build networks; and video conferencing platforms can enable live meetings and presentations, private chats, and breakout rooms for group work ( Dunlap and Lowenthal, 2009). Additionally, there is a growing list of tools to create digital learning content such as interactive images and videos, as well as collaborative platforms that support messaging and content sharing across multiple platforms ( Bates, 2015). Effective integration of digital tools and technologies is facilitated by teachers who explore and learn technologies and new pedagogical practices, as well as the support of leaders, technology support staff, and most importantly, students.

## Tip 3 – Explore strategies to engage with learners virtually

When designing your program, it is important to consider not only content, but also how you plan for learners to engage with that content. Engagement refers to the time and energy expended on learning activities, and includes reading, practicing, obtaining feedback, analyzing the material, and solving problems ( Robinson and Hullinger, 2008). Forging positive connections with instructors has been shown to play a significant role in student satisfaction, persistence, and success ( O’Shea, Stone and Delahunty, 2015). For example, a simple welcome video, recorded on your cell phone and uploaded to the program’s webpage, will go a long way to make your course feel more personalized ( Reyna, 2020). Initiating and maintaining discussions through email and forums, as well as virtual office hours (either a casual drop-in or sign-up for specific time slots) facilitates positive virtual engagement with students. If learners feel welcome to connect with you, they are more likely to seek the answers to their questions. It is important to remember that this form of engagement may take time to grow, however, the development of mutual trust will increase the effectiveness of online collaborative learning ( Sanders, 2013).

## Tip 4 – Design educational content for virtual environments

Consider cognitive load theory (CLT) in course design for remote instruction during COVID-19, as increased stress during the pandemic may have an additional negative impact on working memory ( Klein and Boals, 2001; Hubbard and Blyler, 2016). CLT posits that three types of information ‘load’ processing influence learning: intrinsic load, extraneous load, and germane load, and that these loads impact working memory ability to effectively manage the learning task ( Young *et al.*, 2014). Specifically, CLT is based on the premise that we have limited working memory space, so the aim is to maximize use of working memory by minimizing extraneous load and optimising germane load. Teachers can accomplish this by removing things that distract from the learning process (for example, images that do not relate to the learning goal), selecting an appropriate level of intrinsic load (i.e. make the learning appropriate to the level of the learning) and maximizing germane load (those tasks associated with learning processes). For remote instruction and teaching in virtual environments, we suggest focusing on strategies that reduce extraneous load and optimize germane load. Teachers can reduce extraneous load in a number of ways:

• Most instruction should have both written and verbal explanations in a single integrated source, especially for novices ( Tindall-Ford, Chandler and Sweller, 1997). When integrating verbal with written, avoid splitting students attention between multiple windows on the screen (e.g. chat box, video stream, slides, whiteboard, etc.) ( Ayres and Sweller, 2005; Chen and Wu, 2015).
• Providing worked examples, whether showing the learner how a problem is solved or giving the learner a sense of what the desired end product looks like ( Van Merriënboer and Sweller, 2010).

Teachers can enhance germane load by planning activities that focus on retrieval practice, that is, retrieving the learned information to apply it. In a virtual environment, recommendations for this include

• Allowing students to ‘self-explain’ the material being presented, or summarize key points, whether verbally, or by using the chat feature available in many tech solutions. Breakout rooms can also be used to have students self-explain content to small groups.
• When using active learning actitivities ensure adequate variability in them, so solving each case is a unique exercise of retrieval practice.

## Tip 5 – Consider using diverse teaching strategies

The affordances of new media provide us with opportunities to engage with our learners in new ways and enable meaningful interaction with the content, the instructor, and other learners, as well as the ability to access information and lectures anywhere. However, it is important to be mindful of which technologies your remote students have access to. For example, do they have internet connections to support synchronous technologies, such as break-out rooms, video conferencing, chat areas, and collaboration software? If not, then consider using asynchronous technologies that also facilitate engagement and communication, such as social media (Twitter, YouTube), email, and discussion forums.

Using a variety of learning technologies will not only help you to appeal to different student learning preferences, it can also help you maximise their engagement, and increase collaborative learning. Learning technologies give us the important opportunity of moving our learners from a Web 1.0 environment where they are the consumers of online content to a Web 2.0 environment where they are responsible for creating online content (e.g. blogs, videos, webinars, wikis) (Sandars, 2013). This advantage may be considered an added or extra benefit of virtual course design.

## Tip 6 – Maximise what the technology can do for you

While remote or online learning may seem like an impediment to blend assessment with learning activities, it can also offer an opportunity to consider practices which may be difficult to implement in a larger lecture hall with fixed furniture. For example, punctuating an online lecture with breakout rooms where learners collaboratively complete quizzes (these quizzes may or may not be graded), which are then de-briefed as a large group, can offer valuable and timely formative feedback and assessment for both the learner and teacher. In addition, breakout rooms during an online lecture can also facilitate developing and maintaining learner relationships. This example can also be extended to an asynchronous environment, where the lecture can be provided as a video, the quizzes are completed individually or by groups of learners offline, or through discussion forums, and then debriefed as a scheduled lecture, or follow-up video based on learner achievement in the quizzes. Providing these kinds of assessments also facilitates opportunities for learners to self-assess their knowledge, and adjust their preparations for the scheduled lecture, so that they can maximise their learning. Teachers can connect follow-up resources to specific test questions to assist in this preparation as well, which can be provided through the learning management system.

## Tip 7 – Determine the purpose of assessment and ensure alignment

When moving to online learning environments the purpose of assessments should be forefront. Assessments should reflect the intent, level of mastery, and depth of understanding required to achieve the learning objectives. Moreover, consideration should be given to whether the assessments are providing formative feedback or a summative grade, and if they are realistically achievable online. In this respect, the online setting will only change the format but not the substance of an assessment. Authentic assessments, which mirror workplace contexts where learners apply their knowledge ( Beck and Hatch, 2010), are engaging options for virtual environments, mitigate against plagiarism, and facilitate formative feedback ( Villarroel *et al.*, 2018). The use of authentic and other formative assessments in online contexts also facilitates greater interaction and engagement with the delivery of the feedback through online discussions, peer reviews using shared documents, written feedback, synchronous or asynchronous video.

## Tip 8 – Refine assessments to reflect virtual environments

If changes to assessments are needed with the shift to online delivery, it is important to remember that there is a consistent need for learners to receive quality and timely feedback to enhance their learning experiences ( Reyna, 2020). Therefore, when considering what changes need to occur in a COVID-19 impacted environment, it is valuable to consider the level of mastery that each assessment addresses, in addition to the format of the assessment ( Amin, Shehata and Ahmed, 2020). One way to gauge whether assessments need to be refined is to develop an assessment map, where learning objectives are listed and a link to each assessment is articulated. Each assessment can then be viewed to see if it can continue as-is in a virtual environment, or if refinement is needed. Refinements for virtual environments may include combining or splitting assessments to ensure that they cover the necessary level of mastery for each learning objective, as well as providing an opportunity for the learner to scaffold throughout the course. This assessment map provides a visual check of ensuring that each learning objective has an opportunity to appear within an assessment so that the learner has an opportunity to build and demonstrate their level of mastery, as the course progresses in the virtual environment.

## Tip 9 – Familiarise yourself and your students with virtual teaching environment beforehand

It is important to orient yourself and your students with the technology and software encompassing the virtual teaching environment prior to the start of the course. We agree with MacLeod *et al.* (2019) in that orientation materials should be prepared in a manner that facilitates a straightforward working knowledge of the chosen software in a just-in-time manner. These materials should be easily accessible, such as a one-page document, short video, or short podcast. Instructors are suggested to play with the technology in an environment that is conducive to making mistakes and exploring the software’s features prior to the start of the course ( Kanhadilok and Watts, 2014). This exploration will facilitate the technological competency needed to lead the course and prepare the instructor for unanticipated challenges. We also suggest that the orientation materials be distributed to students ahead of time, with encouragement for them to also play with the technology beforehand. Moreover, we advocate for using the first 10-15 minutes of the initial lecture to go through the orientation materials with the students, to make sure that everyone is on the same page and address any questions and concerns.

## Tip 10 – Keep the virtual teaching environment safe and respectful

MacLeod *et al.* (2019) highlight several unanticipated issues with teaching in virtual environments, categorising them as visual, curricular, and auditory exposures. These issues revolve around a different and unique set of social circumstances entailed through video conferencing and virtual environments, specifically people feeling exposed and uncomfortable with their persistent onscreen image, facilitators and support staff being exposed to sensitive teaching materials, and hearing remarks unintended for the environment. To offset these potential exposures, we suggest including a section in the orientation materials discussed above that explicitly discusses common issues associated with visual, curricular, and auditory exposures and raises awareness for both the instructor and the students. Further, we suggest an open and frank discussion of etiquette to establish standards and protocols for interacting within the virtual environment. This discussion should take place at the onset of the course, as well as being outlined in the orientation materials, and should facilitate a respectful teaching environment ( Terry, Taylor and Davies, 2019). Common etiquette suggestions include muting the microphone unless you are speaking, using the raise hand feature in video conferencing software to indicate that you would like to speak, and clarifying small issues in the chat feature.

## Tip 11 – Make a back-up plan

Making a back-up plan is sound practice regardless of whether teaching in-person or in a virtual environment. However, teaching in virtual environments may increase the potential for unknown and unpredictable factors impacting on the lesson, as instructors and students are both heavily reliant on technology to facilitate communication. Being familiar with the technology and software used to deliver the course may facilitate greater flexibility and adaptability if something does go wrong. Consulting with IT specialists ahead of time may also obviate technological issues, as they can advise on common issues and successful workarounds. We also suggest developing strategies and plans if there are issues with technology (software, internet connection, hardware, etc.). For example, if technological issues impede a virtual lecture, consider shifting to a flipped classroom approach ( Tolks *et al.*, 2016; Hew and Lo, 2018), where the materials can be provided to the students outside of the lecture and discussion can resume once the issue has been resolved. We also suggest considering flexibility in assessment practices, and potentially including independent assignments, as they may be more resilient to technological issues than online real-time assessments ( Rachul *et al.*, 2020).

## Tip 12 – Maintain understanding and compassion for students with the transition to remote learning

Stress is already a major component of student life throughout health professions education ( Ang and Huan, 2006; Bedewy and Gabriel, 2015), and has increased with the COVID-19 pandemic ( Cao *et al.*, 2020; Sahu, 2020). In this respect, it is important to maintain compassion and understanding for students who may be experiencing increased anxiety, depression, and stress, related to exposure to COVID-19 (themselves, friends, family), self-isolation, lack of employment and/or funding, for international students who may need to leave the country, or some combination of the above. Research suggests that social supports and self-compasssion, that is being able to recognise that failures and disappointments, are important for increasing resilience to stress and anxiety ( Neff, Hsieh and Dejitterat, 2005; Poots and Cassidy, 2020). Social supports within remote learning contexts can be facilitated through regular group study assignments that encourage small group actions and cooperation, and may lead to socialisation outside of the course. Self-compassion is more challenging to encourage in virtual environments, but may be developed through more frequent and lower stakes assessments and/or self-assessment. Above all, maintaining compassion and understanding encompasses making time and space to listen to student concerns and being flexible in response to their unique situations and contexts.

## Conclusion

The onset of the COVID-19 pandemic has forced a rapid shift to virtual teaching environments. Our tips are designed to facilitate this rapid shift in health professions education, as well as in the broader education community. In this respect, these tips reflect on the practice of education, commenting on course preparation and development, engaging with students, designing relevant assessments, and working with, rather than against, the technology. We also see these tips as a starting point for shifting from traditional face-to-face lecturing to virtual environments, strengthening considerations of virtual teaching environments as viable and attractive educational tools, and encouraging further discussion of techniques and strategies that contribute to successful virtual classrooms in the health professions education.

## Take Home Messages

• Consider how available technologies can facilitate course delivery in virtual environments, while maintaining course objectives.
• Employ teaching strategies grounded in learning theory that is germane to virtual teaching environments, such as cognitive load theory.
• Refine assessments to reflect best practices in virtual environments and maintain alignment with learning objectives.
• Be mindful of students and maintain compassion, as they are also transitioning to virtual education environments.

## Notes On Contributors

**Benjamin Collins:** M.A., Ph.D., is a Research Associate with the Office of Educational & Faculty Development and an Adjunct Assistant Professor with the Department of Anthropology at the University of Manitoba. ORCiD: https://orcid.org/0000-0002-2416-3184 Twitter: @BenRCollins

**Mr. Ryan Day:** M.E.T., is an Educational Specialist with the Office of Educational & Faculty Development, Rady Faculty of Health Sciences, University of Manitoba.

**Ms Joanne Hamilton:** RD, B.H.Ec., M.Ed., EdD(c), is the Director of the Office of Educational & Faculty development, and an Assistant Professor with the Rady Faculty of Health Sciences, University of Manitoba. Twitter: @JoH_MedEdExplr

**Ms Kathleen Legris:** M.Ed., is an Educational Specialist with the Office of Educational & Faculty Development, Rady Faculty of Health Sciences, University of Manitoba. Twitter: @kathleenlegris

**Dr. Helen Mawdsley:** B.Sc., MAL(H), EdD, is an Educational Specialist and Assessment & Evaluation Lead with the Office of Educational & Faculty Development, and an Assistant Professor with the Rady Faculty of Health Sciences at the University of Manitoba.

**Ms Tanya Walsh:** M.E.T., is an Educational Specialist with the Office of Educational & Faculty Development, Rady Faculty of Health Sciences, University of Manitoba. Twitter: @TanyaCWalsh
