# Supplementary material for: 12 Tips for Pivoting to Teaching in a Virtual Environment
Source: MedEdPublish (2016). 2020 Aug 18;9:170. [Version 1] doi: 10.15694/mep.2020.000170.1 (PMC10702650; doi:10.15694/mep.2020.000170.1)
Supplement: Peer review history

# Reviewer report: Reviewer response for version 1

**DOI:** 10.21956/mep.20174.r31187

**Published:** 2020-08-26

**Authors:** P Ravi Shankar

**Affiliations:** 1 American International Medical University

This review has been migrated. The reviewer awarded 4 stars out of 5 This is an interesting manuscript which will be very helpful to faculty members who are struggling with the rapid transition to online learning during COVID-19. The article sums up and brings together strategies which have been mentioned in other articles published in the journal. Shifting to online learning has a number of challenges. I would be interested in knowing how faculty at the University of Manitoba are engaging with learners virtually and what changes to assessments have been carried out in line with the virtual environment. Health professions educators may be facing greater and particular challenges due to the restrictions on patient contact and on clinical learning. More tailored suggestions and tips for this group of educators will be very helpful.

Reviewer Expertise:

NA

No decision status is available

# Reviewer report: Reviewer response for version 1

**DOI:** 10.21956/mep.20174.r31185

**Published:** 2020-08-25

**Authors:** Alexandra Webb

**Affiliations:** 1 Australian National University

This review has been migrated. The reviewer awarded 3 stars out of 5 Thank you for sharing these practical tips based on your institutional experience of transition to a virtual environment during COVID-19. They provide a comprehensive and useful reference for this disruption that continues to impact health profession education.Generally, the article would benefit from positioning the tips in the context of health profession education. This should include the description of examples from your institutional experience and citations from the health profession education literature.Specific points that it would be useful to elaborate on include:Tip 1 – what about learning outcomes that cannot be aligned with the virtual environment? This is an especial challenge in health professions education for a range of practical activities that it may not be possible to transition to a virtual setting but are essential for safe and competent practice and meeting accreditation standards e.g. physical examination, diagnostic proceduresTip 4 – how did you ensure that in the transition to a virtual environment the educational design also considered requirements for accessibility to meet the needs of students with physical or cognitive impairments?Tip 5 – a potential risk of using too many different learning technologies can be that students don’t know where to find relevant information at the time that they need it or understand where it fits within the curriculum. How did you address this at your institution to ensure that students could locate information available on different platforms and clearly identify where it belonged in the curriculum?Tip 9 – given the very sudden disruption at many institutions, with no or limited notice to transition to a virtual environment, how do you advise being able to achieve this at short notice?

Reviewer Expertise:

NA

No decision status is available

# Reviewer report: Reviewer response for version 1

**DOI:** 10.21956/mep.20174.r31189

**Published:** 2020-08-25

**Authors:** Tharin Phenwan

**Affiliations:** 1 University of Dundee

This review has been migrated. The reviewer awarded 3 stars out of 5 Thank you for your practical tips that we all need in this unusual time.Apart from previous feedback, I would like to add more on:-Tip 1: could you elaborate further on dealing with learning objectives that may not align well with virtual environment such as practical skills? It is extremely challenging to monitor practical sessions with the students and give them constructive feedback and appropriate support. Some students may not have someone else to practice their practical skills or lack appropriate equipment to be fully engage with the learning materials.-Tip 5: can you explain more as in how does your institute mitigate this issue? From what I found in my place, students and tutors are struggling to keep up with several software that we need to use in our module.These tips will be of use to healthcare instructors who need to teach online.

Reviewer Expertise:

NA

No decision status is available

# Reviewer report: Reviewer response for version 1

**DOI:** 10.21956/mep.20174.r31186

**Published:** 2020-08-23

**Authors:** Megan Anakin

**Affiliations:** 1 University of Otago

This review has been migrated. The reviewer awarded 3 stars out of 5 I read this Practical Tips article with interest because we have had to move rapidly into online learning environments with the clinical educators at my university during the COVID19 pandemic. I was particularly interested in how the suggestions and insight gained by the authors in a Canadian health professions education context could be applied to my situation in New Zealand. I have a few suggestions that might help to enhance your article. For example, to help the reader appreciate Tip 1, please consider giving an example of how a health professions education learning outcome was aligned to a virtual environment in your institution and how others may have achieved this goal by citing relevant health professions literature. For Tip 2, please consider providing an example of a particular tool that an educator at your institution used to create digital learning content for a group of health professional students and other examples of tools used by other health professions educators in the literature. For Tip 3, please consider addressing some engagement strategies that might be unique to a student in, and a feature of, health professions learning environments. As with the other two tips, there might be articles in the health professions education literature that can be cited to provide examples from contexts beyond your institution to help international readers appreciate how the strategies you suggest might apply to their own contexts. There may be important cultural and technological differences between your experiences and those of educators elsewhere in the world that could be signposted to help readers think about how they might apply each of the 12 tips to their situations.To enhance the take home messages for health professions educators, please consider how you might enhance the relevance of the four points to this group of educators because they may be facing particular issues and constraints because they may be teaching in a workplace learning environment that has been severely impacted by caring for COVID19 patients.

Reviewer Expertise:

NA

No decision status is available

# Reviewer report: Reviewer response for version 1

**DOI:** 10.21956/mep.20174.r31188

**Published:** 2020-08-19

**Authors:** Felix Silwimba

**Affiliations:** 1 University of Lusaka

This review has been migrated. The reviewer awarded 4 stars out of 5 The paper is about transitioning to online medical education in the covid-19 era.Strengths of the paper:• It is original and current.• The title is consistent with the contents of the manuscript.• The abstract has correctly summarized the contents.• The background has provided sufficient to read on the manuscript.• The 12 points are well thought through and comprehensive.• The references are recent.Weakness of the paper:In points 5 and 6 it could have been better to clearly explain the use of recorded media such as CDs and flash drives, including static websites dedicated to medical education. I think this will make learning broadly appealing to all regions.

Reviewer Expertise:

NA

No decision status is available
